# Aspergilloma in an Immunocompetent Host: A Case Report

**DOI:** 10.7759/cureus.40727

**Published:** 2023-06-21

**Authors:** Hamad AlShaheen, Yacoub Abuzied, Hosam Aldalbahi, Mohammed AlSheef

**Affiliations:** 1 Infectious Disease and Internal Medicine, Medical Specialties Department, King Fahad Medical City, Riyadh, SAU; 2 Nursing Department, Rehabilitation Hospital, King Fahad Medical City, Riyadh, SAU; 3 Internal Medicine, Medical Specialties Department, Main Hospital, King Fahad Medical City, Riyadh, SAU; 4 Internal Medicine and Thrombosis, Medical Specialties Department, King Fahad Medical City, Riyadh, SAU

**Keywords:** pulmonary aspergillosis, pulmonary, immunocompetent, aspergillus niger, aspergilloma

## Abstract

Aspergillosis is a serious pathologic condition caused by *Aspergillus* that commonly affects immunocompromised patients. In recent years, it has been demonstrated that *Aspergillus* infection can cause a wide spectrum of pulmonary diseases, including allergic bronchopulmonary aspergillosis, chronic necrotizing aspergillosis, aspergilloma, and invasive aspergillosis. The characteristic computed tomography (CT) and pathologic findings of the various pulmonary manifestations of *Aspergillus* infection are illustrated and reviewed in this case report.

*Aspergillus niger* is an infrequent infection that affects the lungs in severely immunosuppressed patients. In this paper, we report the case of a 50-year-old female with well-controlled type 2 diabetes mellitus who presented to the emergency department with a history of shortness of breath, cough, and weight loss. She denied any use of immunosuppressive medications. High-resolution CT revealed a large right upper lung cavitary lesion, and the sputum examination and bronchoalveolar lavage revealed *Aspergillus niger* and positive *Aspergillus* galactomannan. In conclusion, immunocompetent hosts are rarely affected by aspergilloma with lung cavities. We recommend conducting a retrospective data registry on unreported aspergilloma cases in immunocompetent patients to understand the clinicopathological behavior and improve management.

## Introduction

The lung cavity is a gas-filled space within a zone of pulmonary consolidation or within a mass or nodule produced by the expulsion of a necrotic part of the lesion via the bronchial tree [1]. The pathophysiology of the lung can be due to many processes, such as suppurative necrosis, infarction (ischemic necrosis), cystic displacement of lung tissue, and malignant tumor desquamation [1]. *Aspergillus* causes various diseases, including pulmonary aspergillosis, rhinosinusitis, central nervous system aspergillosis, endocarditis, osteomyelitis, and endophthalmitis [2]. *Aspergillus fumigatus* is one of the most predominant airborne saprophytic fungi worldwide [3]. Its natural ecological niche is the soil, where it survives and grows on organic debris [4]. Several forms of presentations exist, including aspergilloma, allergic bronchopulmonary aspergillosis, chronic necrotizing (formerly semi-invasive) aspergillosis, and invasive aspergillosis [5]. *Aspergillus* *fumigatus* is also present in indoor environments, including hospitals [6].

*Aspergillus* *fumigatus* has become the most prevalent airborne fungal pathogen causing severe and fatal invasive infections in immunocompromised hosts in developed countries [7]. Other species of *Aspergillus* *niger* are associated with otomycosis and cutaneous infection, and it is rarely associated with lung infection [8]. To our knowledge, aspergillosis causes lung cavity and invasive aspergilloma in immunocompromised hosts. Here, we present a particular case of aspergillosis and lung cavity in an immunocompetent host.

## Case presentation

A 50-year-old female with well-controlled type 2 diabetes mellitus (T2DM), on the oral hypoglycemic agent metformin with HbA1c 5.9 mmol/L, presented to the emergency department during the COVID-19 pandemic with a history of shortness of breath and cough for three days associated with a sore throat, runny nose, and weight loss of approximately 5 kg in one month. She denied any history of hemoptysis, use of immunosuppressive medications, chest pain, recent contact with a sick patient, exertional dyspnea, or paroxysmal nocturnal dyspnea.

There was no past medical history of asthma, steroid use, or chronic lung disease. Examination revealed decreased air entry on the right side and hyper-resonance in the right lower lobe. The cardiac examination and other systems were unremarkable. Blood pressure was 126/70 mmHg, axillary temperature was 37.1°C, blood glucose was 10.5 mmol/L, oxygen saturation was 94% on a 6 L simple face mask, and heart rate was 96 beats per minute.

The initial lab findings showed acute kidney injury, serum creatinine of 245 µmol/L, and urea, and other biochemistry labs were normal. The complete blood count revealed a high white blood cell count (16.58 × 10^9^/L), neutrophilia of 79%, and normal hemoglobin and platelet counts (Table 1).

**Table 1:** Laboratory results of the patient at the initial visit.

Laboratory test	Result	Reference range
White blood cell count	16.58	3.9–11 × 10^9^/L
Neutrophils	79%	30–70%
Absolute neutrophil count	13.19	1.35–7.5
Hemoglobin	11.5	11–16 g/dL
Platelet count	385	155–435 × 10^9^/L
Creatinine	245	64–104 µmol/L
Urea	19	2.5–6.7mmol/L
Sodium	135	136–145 mmol/L
Alanine transaminase	27	0-55 U/L
Hemoglobin A1c	5.9	5.7–6.5 mmol/L
Albumin, plasma	34	35–32 g/L
Human immunodeficiency virus 1 and 2	Negative	
Anti-hepatitis C virus	Negative	
Hepatitis B surface antigen	Negative	
Hepatitis A virus	Negative	
Antinuclear antibody	Negative	
Potassium	3.24	3.5–4.5 mmol/L
Alkaline phosphatase	165	40–150 U/L
Bicarbonate	18	23–28 mmol/L
QuantiFERON-Tb Gold	Indeterminate	Negative
Legionella antigen detection, urinary antigen test	Negative	Negative
Aspergillus galactomannan	0.884	<0.5
Fungal examination sputum	Aspergillus niger	
Sterile body fluid for sputum, bronchoalveolar lavage	Aspergillus niger	
Mycobacterium examination sputum	Negative	
Viral respiratory polymerase chain reaction panel	Negative	
QuantiFERON-Tb Gold	Negative	
IgA	4.79	0.7–4.0 g/L
IgM	0.74	0.4–2.3 g/L
IgG	14.3	7.0–16.0 g/L
Antinuclear antibodies	Negative	
Antineutrophilic cytoplasmic antibody	Negative	

**Figure 1:**
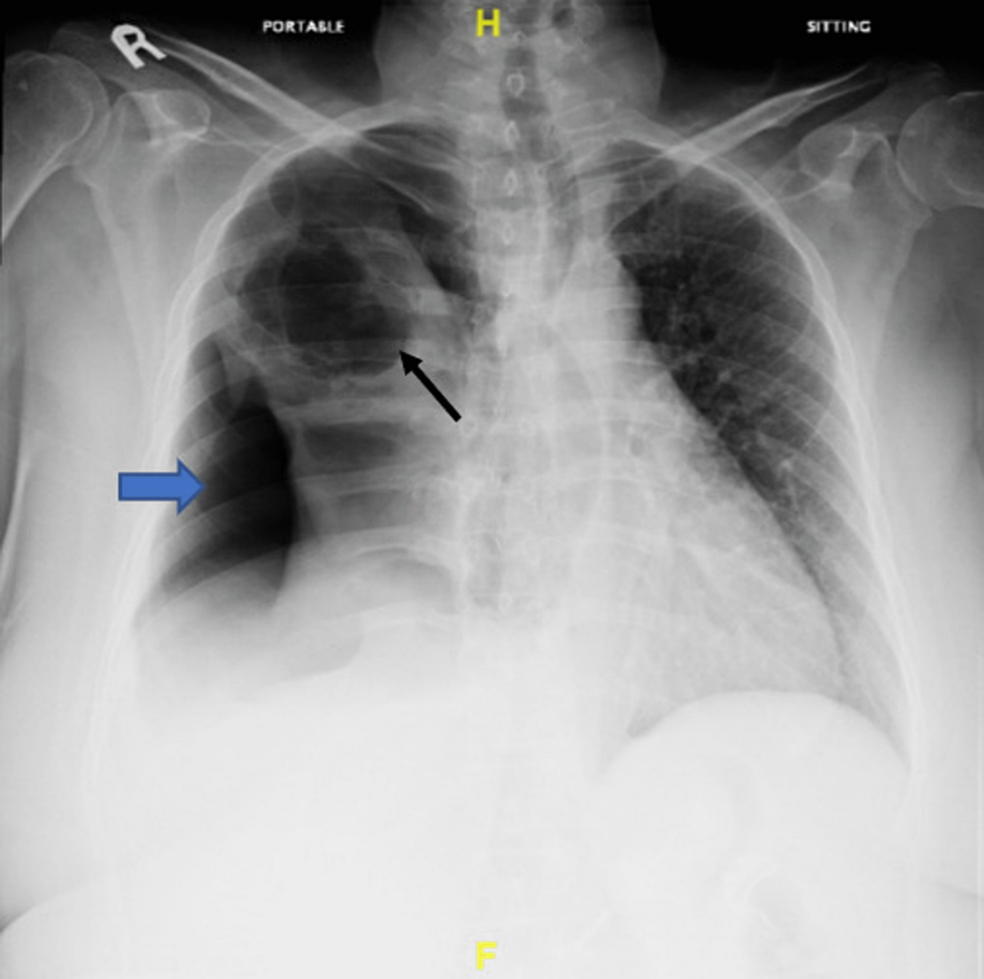
Chest radiograph showing right hydropneumothorax with the complete collapse of the right lung with a large cavitary lesion.

A chest radiograph revealed a right hydropneumothorax with the complete collapse of the right lung (blue arrow) and a large cavitary lesion in the right upper lobe (arrow line). A slight shift of the mediastinum to the left was observed (Figure 1).

**Figure 2:**
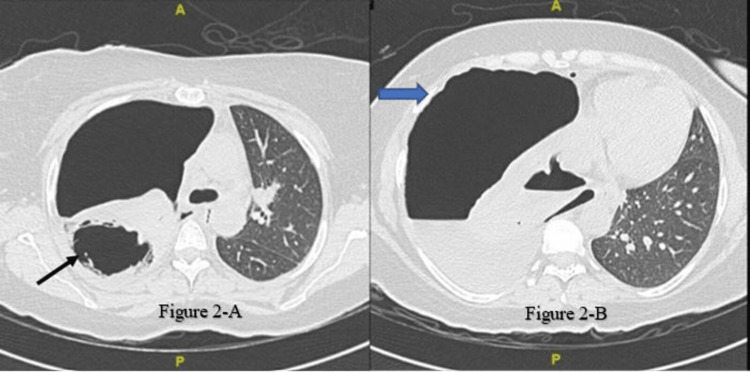
High-resolution computed tomography of the lungs showing a large right-sided hydropneumothorax with a mediastinal shift to the left side, complete collapse of the right lung, and narrowed right-sided bronchial tree (blue arrow). Large right upper lung cavitary lesion right upper lung large cavitary lesion measuring 4.7 × 6.5 × 6.8 cm can be seen (arrow).

At admission, the patient received piperacillin/tazobactam at a renally adjusted dose of 2.25 g intravenously every six hours for 14 days. High-resolution computed tomography (CT) revealed a large right-sided hydropneumothorax, a mediastinal shift to the left, a complete collapse of the right lung, and a narrowed right-sided bronchial tree. The trachea and bronchial tree of the left lung were visible. A large right upper lung cavitary lesion measuring 4.7 × 6.5 × 6.8 cm was discovered (Figures 2B, 2B).

**Figure 3:**
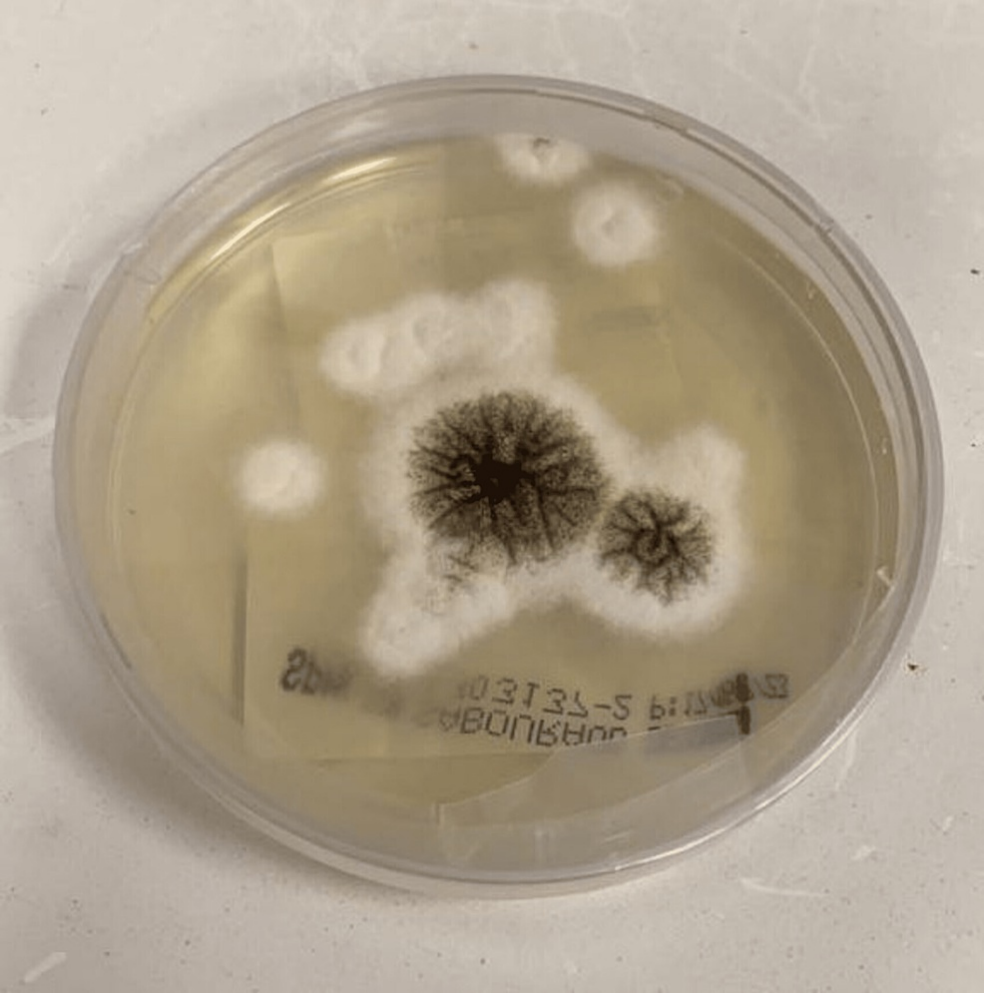
Fungal culture shows septated, hyaline hyphae, smooth conidiophores, biseriate phialides, and brown conidia in chains.

As the sputum examination revealed *Aspergillus niger*, the patient underwent bronchoalveolar lavage, which revealed *Aspergillus niger* and positive *Aspergillus* galactomannan at 0.884. Fungal culture revealed septated and hyaline hyphae. Conidiophores were long and smooth. Biseriate phialides radiated from large vesicles. Conidia were brown and occurred in chains, which is typical with *Aspergillus niger* (Figure 3).

Acid-fast bacilli were not found in the sputum. The HIV test came back negative, as did the immunological workup for antinuclear antibodies. An immunocompetent host was diagnosed with aspergilloma. Based on the infectious diseases consultation, the patient was started on voriconazole intravenously for six weeks as an antifungal treatment, which was initiated after the renal impairment was normalized. The leukocytosis improved after the antifungals were finished, but the symptoms persisted. Thoracic surgery was also consulted, and chest tubes were inserted into the right lung drainage, and the pneumothorax was evacuated.

**Figure 4:**
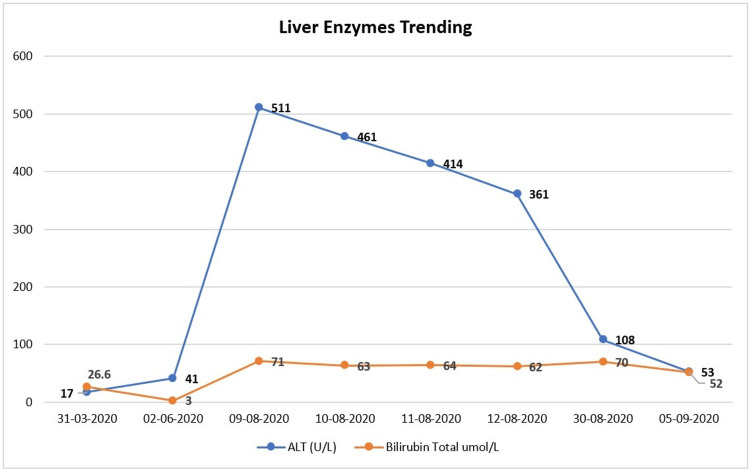
The trend of liver enzymes.

During follow-up, the patient was noticed to have elevated liver enzyme which during hospitalization was normal. After six months of diagnosis, the patient’s liver enzymes, alanine aminotransferase and direct bilirubin, were increasing, and trending in stationary elevation for one month then started to decline (Figure 4).

Finally, the patient was discharged on voriconazole oral tablets for eight weeks. Follow-up appointments with thoracic surgery and infectious diseases were scheduled for further planning and management; however, the patient did not return for follow-up. We attempted to communicate with the patient via phone numerous times but received no response. This could be due to the COVID-19 pandemic and lockdown.

## Discussion

Aspergillosis is an infection caused by *Aspergillus*, a common mold (a type of fungus) that lives both indoors and outdoors [9]. Most people inhale *Aspergillus* spores on a daily basis without acquiring an infection. People with compromised immune systems or lung diseases comprise patients with prolonged neutropenia, allogeneic hematopoietic stem cell transplant, solid organ transplant, inherited or acquired immunodeficiencies, corticosteroid use, and others who are more likely to acquire *Aspergillus*-related health problems [10]. *Aspergillus *causes allergic responses, lung infections, and infections in other organs [11]. In the presence of invasive aspergillosis, the spores grow into hyphae, which enter blood vessels and produce hemorrhagic necrosis and infarction [12]. Asthma, pneumonia, sinusitis, or a rapidly progressing systemic infection may cause symptoms. Imaging, histology, specimen staining, and culture are all diagnostic tools. Voriconazole, amphotericin B (or its lipid formulations), caspofungin, or itraconazole are used to treat this condition. Fungus balls may necessitate surgical excision.

Clinical presentation (symptoms and signs), comorbidities such as T2DM, investigations (lab and radiology), treatment response, and follow-up were compared in the local and international literature, along with *Aspergillus *and the relationship between diabetes control and the risk of infection with aspergilloma, disease complications, and antifungal therapy (drug-induced hepatitis). Pulmonary disease is most commonly caused by *Aspergillus fumigatus*, while other diseases can be caused by species such as *Aspergillus flavus*, *Aspergillus niger*, and *Aspergillus terreus* [13]. The clinical spectrum of aspergillosis ranges from aspergilloma to fatal invasive disease. Clinically distinct aspergillosis is one of the four major entities: (i) allergic bronchopulmonary aspergillosis, which afflicts patients with long-standing asthma; (ii) chronic necrotizing aspergillosis or semi-invasive aspergillosis (old classification), which afflicts patients with a history of chronic lung disease; (iii) aspergilloma, which primarily affects patients with pre-existing lung cavities; and (iv) invasive aspergillosis, which afflicts immunocompromised and critically ill hosts [14,15].

The air crescent sign is classically described in pulmonary aspergillosis either with an aspergilloma in a pre-existing lung cavity or during the recovery phase of invasive aspergillosis when air fills the space between the retracting devitalized infected lung tissue and the parenchyma. However, the air crescent sign is not specific and can be seen in other infections such as melioidosis [16]. Chronic necrotizing aspergillosis or semi-invasive aspergillosis is characterized by an indolent course of constitutional symptoms such as weight loss, malaise, and fatigue, as well as cough and occasionally hemoptysis [7,17]. *Aspergillus niger* is a very uncommon infection, in contrast to *Aspergillus fumigatus*, which is the most common organism that causes lung infection [2]. *Aspergillus niger* was reported in severely immunosuppressed patients, such as patients on high doses of steroids, lung transplants, bone marrow transplants, and severely neutropenic patients (post-COVID-19 pneumonia and post-treatment with tocilizumab) [18]. In contrast, our patient presented with progressive weight loss and cough, a lung cavity with the absence of lung disease and immunosuppression, and her diabetes was controlled for hemoglobin A1c. Finally, in immunocompetent patients, *Aspergillus niger* was not commonly found to cause aspergilloma and induction of the lung cavity. Following the updated and concerned guidelines will lead to a reduction in hospital stays, facilitating financial, operational, and clinical outcomes [19].

In our case, the patient developed a significant burden of aspergilloma, with complications including pneumothorax, requiring pigtail insertion in the affected side by a thoracic surgeon. The aim was to start intravenous antifungal therapy for six weeks and then follow up in the outpatient thoracic surgery clinic for possible surgical intervention, as preoperative antifungals can be started in such cases to prevent the possibility of surgical spilling of the aspergilloma during surgical resection and subsequent *Aspergillus* empyema according to the clinical practice guidelines updated by the Infectious Disease Society of America, 2016 [20].

## Conclusions

Aspergilloma is more frequently seen in immunocompromised patients, such as those with extended neutropenia, allogeneic hematopoietic stem cell and solid organ transplant, hereditary and acquired immunodeficiency conditions, corticosteroid use, and others; however, it can also occur in immunocompetent hosts. The differential diagnosis of lung cavities in immunocompetent patients should include more common etiologies such as bacterial, pulmonary tuberculosis, and connective tissue disease in addition to aspergilloma. Although surgical resection is considered the standard of care for solitary aspergilloma if no contraindication is present, peri or postoperative antifungal therapy with voriconazole is required if the risk of surgical spillage of the aspergilloma is high to prevent *Aspergillus* empyema. We recommend conducting a retrospective data registry on unreported aspergilloma cases in immunocompetent patients to understand the clinicopathological behavior and improve management.
